# Medical Journals and Patent Medicines

**Published:** 1871-01

**Authors:** 


### MEDICAL JOURNALS AND PATENT MEDICINES.

Medical journals profess great hatred for all manner of proprietory medicines, and take every possible occasion to abuse them as well as their proprietors. But we notice, in almost every journal that comes to our table, they do not refuse to receive their advertisements and money. It is true, they do not countenance advertisements of Dr. ———'s Golden Female Pills," or "Mrs. Winslow's Soothing Syrup"; but they *do* receive and print notices of so-called remedies, that smack quite as much of humbuggery. We see in many respectable medical journals, as well as upon sundry highly respectable bill-boards and lamp-posts, the names of marvelous "Pectorial Pasteles"—Iodo-Ferro-Phosphated-Elixir-of-Horse-Radish"—"Blanchard's Pills,"—"Golden-Cod-Liver-Oil, with hypophosphite of Lime," "——'s Electric Disk,—a sure cure for rheumatism." Pills of all sorts and kinds, recommended for all manner of ailments, by what are supposed to be respectable physicians.

For the life of us, we cannot see why these proprietory medicines and appliances have any more claim for recognition from the profession, than the numerous "Bitters" that we find posted in the secular press and on our mountain tops. Perhaps the fact that the remedies advertised through the medical press, are supposed to contain ingredients which are made known to the profession, is considered sufficient excuse for their appearance. If so, we doubt not that "Mrs. Winslow," "S. T. 1860 X," and all the rest of the patent medicine men, would be willing to give their formulæ, also, for the benefit of an extensive circulation through the medical press.

The truth is, proprietory pills, capsules, lotions, cod liver oil, etc., are becoming entirely too common, and are too fully countenanced by Physicians and Medical Journals. So profitable has it become to manufacture something of this sort, that there is not to-day, a wholesale druggist or chemist in America, that does not manufacture some confounded pill or other, and seek to palm it off upon respectable physicians as a specific for some manner of disease. At the rate they are being manufactured, at no distant day, the practice of medicine promises to be entirely in the hands of these enterprising druggists, who will have a pill for every disease known to Da Costa's Diagnosis. We have too much confidence in the intelligence of the medical profession to suppose that they purchase all these abominable nostrums,—somebody must buy them, else there would be no such demand for them. It is reasonable, therefore, to suppose, that their sale is effected through the advertising of medical journals, assisted by posters and circulars, and that non-professional readers are the heaviest purchasers.

Gentlemen of the Medical Press, "let up"—no more patent-pill advertising. Be consistent, do not exclude one class of patent medicines, and laud another. Expunge them all, and devote the space so occupied to common sense and legitimate medicine.

